# The impact of the donors’ COVID-19 status on the outcomes of allogeneic hematopoietic stem cell transplantation: a multi-center retrospective study

**DOI:** 10.3389/fmicb.2024.1415289

**Published:** 2024-07-15

**Authors:** Yifei Huang, Zhiping Fan, Yingying Hu, Sizhou Feng, Shunqing Wang, Shanyu Zhang, Fen Huang, Li Xuan, Na Xu, Hui Liu, Zhixiang Wang, Jing Sun, Qifa Liu, Ren Lin

**Affiliations:** ^1^Department of Hematology, Nanfang Hospital, Southern Medical University, Clinical Medical Research Center of Hematological Diseases of Guangdong Province, Guangzhou, China; ^2^State Key Laboratory of Experimental Hematology, National Clinical Research Center for Blood Diseases, Haihe Laboratory of Cell Ecosystem, Institute of Hematology & Blood Diseases Hospital, Chinese Academy of Medical Sciences & Peking Union Medical College, Tianjin, China; ^3^Department of Hematology, Guangzhou First People’s Hospital, Guangzhou, China

**Keywords:** allogeneic hematopoietic stem cell transplantation, coronavirus-19, donor selection, engraftment, graft-versus host disease

## Abstract

**Introduction:**

To explore the impact of donors’ COVID-19 status on allogeneic stem cell transplantation (allo-HSCT), we compared the transplant outcomes of 74 participants.

**Methods:**

This multi-center retrospective study included nine participants receiving grafts from COVID-19 positive donors (CPD), 45 from COVID-19 experienced donors (CED), and 20 from COVID-19 naive donors (CND). We evaluated engraftment, complications, and survival rates among the three groups.

**Results:**

All apheresis procedures were successful with no significant differences in CD34+ cells or lymphocytes in grafts among the three groups. All patients achieved engraftment by day 30 post-HSCT. The incidence of grade II-IV acute graft-versus-host disease (aGVHD) was 55.6%, 20%, and 10% in the CPD, CED, and CND groups, respectively (*p* = 0.024). Multivariate analysis indicated that COVID-19 positivity in donors at the time of apheresis was an independent risk factor for II-IV aGVHD (*p* = 0.020, OR = 12.159, 95% CI 1.783 -135.760). No differences were observed among the groups in terms of chronic GVHD, viral infection, or sinusoidal obstruction syndrome. The 6-month overall survival and disease-free survival rates were also similar among the three groups.

**Discussion:**

Our results suggest that the COVID-19-positive status of donors might not impact graft collection, engraftment, or short-term survival of allo-HSCT recipients but might increase the risk of aGVHD. Further research is needed to explore the influence of donors’ COVID-19 status on long-term complications and survival in allo-HSCT recipients.

## Introduction

Coronavirus disease 2019 (COVID-19) remains a global health issue with a high prevalence, and causes mortality in immunocompromised populations that include recipients of allogeneic hematopoietic stem cell transplantation (allo-HSCT). In the era of the COVID-19 pandemic, to screen the COVID-19 status of HSCT donors before donation has become a common practice, considering the potentially high risk to both allo-HSCT recipients and donors (Worel et al., 2021). Due to the strict epidemic prevention and control strategies performed before in Chinese Mainland, most of the population were not yet be infected by SARS-COV-2 before Dec 2022. With the new epidemic prevention and control strategies carried out on Dec 8 2022 in China, the number of people with COVID-19 increased rapidly and peaked in late December. During the COVID-19 pandemic, however, unexpected SARS-COV-2 infection in an HSCT donor during mobilization and apheresis makes deferred transplantation or changing to an alternative donor difficult or impossible.

The main concern of transplant from donor with COVID-19 is the possibility of viral transmission through grafts. Several previous studies observed no transmission of SARS-CoV-2 from COVID-19-positive donors (Anurathapan et al., 2020; Lázaro Del Campo et al., 2020; Leclerc et al., 2021; Maakaron et al., 2021; Blandin et al., 2022; Koç et al., 2022; Cheng et al., 2023). In addition, these studies showed that there were no adverse effects on apheresis or chimerism in recipients of SARS-CoV-2-positive HCST donors. Nevertheless, most of these studies are case reports or have a small sample-size. Moreover, there is a lack of research to date discussing the impact of active and past COVID-19 infection in donors on HSCT.

Here, we present data from 74 participants across three transplant centers who underwent allo-HSCT between December 2022 and May 2023. Notably, nine participants received transplant from donors who tested positive for SARS-CoV-2 by reverse transcription PCR (RT-PCR) on throat swabs at the time of cell collection. The outcomes of these recipients were compared with those who received transplants from 45 COVID-19-experienced donors and 20 COVID-19-naive donors. This detailed analysis of these specific cases provides critical insights into the feasibility and safety of using COVID-19 positive donors.

## Methods

### Study design and participants

This is a multi-site, retrospective study conducted in three transplant centers in China including Nanfang Hospital, Southern Medical University, Institute of Hematology and Blood Diseases Hospital, Chinese Academy of Medical Sciences, and Guangzhou first people’s hospital. All the participants in this study received allo-HSCT between December 8, 2022 and May 7, 2023. According to the infection status of the donors at transplant, the participants were divided into COVID-19-positive donor (CPD) group, COVID-19-experienced donor (CED) group, and COVID-19-naive donor (CND) group. CPD were defined as those who tested positive for SARS-CoV-2 during the collection of hematopoietic stem cells (HSC), confirmed through RT-PCR performed on throat specimens. CED group refers to individuals who had contracted COVID-19 before HSC collection, confirmed by at least one positive RT-PCR test, but had fully recovered by the time of collection. Their recovery was confirmed by two consecutive negative RT-PCR tests conducted 24 h apart and the absence of clinical symptoms for at least 14 days. CND were those who had never been infected with SARS-CoV-2 before HSC collection; this status was confirmed by the absence of prior COVID-19 symptoms and consistently negative results from past RT-PCR tests. All recipients were COVID-19 negative tested by nucleic acid amplification test (NAT) on throat specimens (National Health Commission of People's Republic of China and National Administration of Traditional Chinese Medicine, 2023) before starting the conditioning regimen, and donors were COVID-19 negative prior to mobilization. Mobilized fresh bone marrow and peripheral blood stem cell grafts were infused (Wang et al., 2016). The targeted CD34+ cell count was more than 2 × 10^6^ per kg of recipient weight.

Data were retrospectively collected from the participants’ and donors’ electronic medical records including demographic and transplant characteristics, engraftment, transplant-related complications, and survival. The demographic of recipients and donors as well as transplant characteristics were summarized in Table 1.

**Table 1:** Demographics and baseline characteristics of allo-HSCT recipients and donors.

	CPD group (*n* = 9)	CED group (*n* = 45)	CND group (*n* = 20)	*p* value
**Recipients**				
**Diagnosis (%)**				0.463
AML	5 (55.6%)	19 (42.2%)	9 (45.0%)	
ALL	2 (22.2%)	15 (33.3%)	9 (45.0%)	
AA	1 (11.1%)	0	0	
MDS	1 (11.1%)	7 (15.6%)	2 (10.0%)	
Others	0	4 (8.8%)	0	
**Age, median (range), years**	34 (14–54)	44 (15–64)	40 (20–56)	0.522
**Sex (%)**				0.748
Male	5 (55.6%)	22 (48.9%)	12 (60.0%)	
Female	4 (44.4%)	23 (51.1%)	8 (40.0%)	
**Graft type (%)**				0.129
PB	8 (88.9%)	25 (55.6%)	14 (70.0%)	
PB + UCB	1 (11.1%)	20 (44.4%)	6 (30.0%)	
**Donor–recipient relationship (%)**				0.929
Sibling	3 (33.3%)	18 (40.0%)	8 (40.0%)	
Family	6 (66.7%)	27 (60.0%)	12 (60.0%)	
**HLA match (%)**				0.929
10 of 10 HLA match	3 (33.3%)	18 (40.0%)	8 (40.0%)	
1 or more HLA mismatch(es)	6 (66.7%)	27 (60.0%)	12 (60.0%)	
**Conditioning regimen (%)**				0.574
BU-based	8 (88.9%)	32 (71.1%)	16 (80.0%)	
TBI-based	1 (11.1%)	13 (28.9%)	4 (20.0%)	
**ATG for GVHD prophylaxis (%)**				0.299
ATG	6 (66.7%)	30 (66.7%)	17 (85.0%)	
Non-ATG	3 (33.3%)	15 (33.3%)	3 (15.0%)	
**Donors**				
**Age, median (range), years**	35 (17–60)	34 (11–60)	31.5 (9–62)	0.819
**Sex (%)**				0.703
Male	4 (44.4%)	22 (48.9%)	12 (60.0%)	
Female	5 (55.6%)	23 (51.1%)	8 (40.0%)	
**COVID-19 severity (%)**				0.561
Mild	9 (100.0%)	39 (86.7%)	**-**	
Moderate	0	6 (13.3%)	**-**	
**Peripheral blood leukocytes day 0, mean (range), x 10** ^**9**^ **/L**	32.80 (21.02–52.36)	35.55 (18.78–67.86)	37.01 (25.07–52.26)	0.782
**Peripheral lymphocytes day 0, (range), %**	7.00 (2.40–13.20)	9.90 (2.80–14.00)	9.05 (6.50–18.20)	0.167
**COVID-19 NAT in graft**	-	-	Negative	-

CPD, COVID-19-positive donor; CED, COVID-19-experienced donor; CND, COVID-19-naive donor; AML, Acute myeloid leukemia; ALL, Acute lymphoblastic leukemia; AA, Aplastic anemia; MDS, Myelodysplastic syndrome; PB, Peripheral blood; UCB, Umbilical cord blood; HLA, Human leukocyte antigen; BU, Busulfan; TBI, Total body irradiation; ATG, Antithymocyte globulin; and NAT, Nucleic acid amplification test.

### Cell composition analysis in grafts

The percentages of CD34^+^ stem cells, T lymphocyte subsets (CD3^+^, CD4^+^, and CD8^+^), natural killer (NK) cells (CD16^+^, CD56^+^), B lymphocytes (CD19^+^), in samples from peripheral blood mononuclear cells grafts were analyzed by flow cytometry (BD FACSCanto II). The absolute numbers of graft compositions were calculated as the percentages of these cells multiplied by the percentages of lymphocytes multiplied by the total nucleated cell.

### Monitoring of Epstein–Barr virus-DNA and cytomegalovirus-DNA levels in blood

Real-time PCR was performed with Epstein–Barr virus (EBV) and cytomegalovirus (CMV) specific primers and probes, the EBV-DNA and CMV-DNA levels of blood were monitored weekly for 3 months after transplantation (twice a week during the first 3 months after transplantation and in case of DNAemia). Once EBV-DNA or CMV-DNA in the blood was positive, the viral loads would be detected once again the next day (Xuan et al., 2012). Either CMV or EBV viremia was defined as the presence of more than 500 copies viral DNA/mL whole blood twice consecutively (Lin et al., 2019).

### Ethics committee approval

The study was performed in accordance with the Declaration of Helsinki and was approved by the Ethics Committee (Approval No.NFEC-202302-K2). The ethical approval protocol included comprehensive guidelines for data collection, participant confidentiality, and the overall conduct of the study.

### Definitions

Engraftment, relapse, overall survival (OS), and disease-free survival (DFS) were assessed as previously described (Zeng et al., 2021). Acute graft-versus-host disease (aGVHD) was defined according to the 1994 Consensus Conference on Acute GVHD Grading and graded from I to IV (Przepiorka et al., 1995). Chronic GVHD (cGVHD) was graded as mild, moderate and severe according to the related criteria (Jagasia et al., 2015).

### Statistical analysis

Descriptive statistics were used to summarize participant baseline characteristics and determine the distribution and frequency of the variables. Chi-square test or Fisher’s exact test were used for clinical feature and outcome analysis, and Wilcoxon rank-sum test was used to compare continuous variables. The Kaplan–Meier survival curve and log-rank test were used for survival analysis. Univariate and multivariate analyses were performed using logistic regression to identify the risk factors for II–IV aGVHD. All *p* values were two-sided with significance level at α = 0.05. The data are analyzed using SPSS 27.0 (SPSS Inc., Chicago, IL, United States) and the R (version 4.3.1, available online at http://www.R-project.org).

## Results

### Study population

The participants’ and their donors’ baseline and transplant characteristics were summarized in Table 1. The median follow-up was 255 days [interquartile range (IQR) 226–319 days]. Thirty-three (44.6%) participants were diagnosed with acute myeloid leukemia (AML), 26 (35.1%) with acute lymphoblastic leukemia (ALL), 10 (13.5%) with myelodysplastic syndrome (MDS), 2 (2.7%) with mixed-phenotype acute leukemia (MPAL), 2 (2.7%) with myelofibrosis, and 1 (1.4%) with aplastic anemia (AA).

All 74 donors were COVID-19 negative tested by RT-PCT on throat swab samples before mobilization. Forty-five donors had a history of COVID-19 with a median time from diagnosis to mobilization of 54 days (range 20–114). Six of the 45 donors experienced a high fever for more than 3 days during their infection but did not show a decrease in SpO2 or any suspicious findings in CT scans; these cases were classified as moderate COVID-19 infections. The remaining 39 donors, who were classified as mild COVID-19 infections, experienced fever lasting 1–2 days without experiencing shortness of breath. Nine donors suffered mild COVID-19 during apheresis of −1 day (median; range -2-0). The symptoms of COVID in these donors included fever in 7 (77.8%), cough and sore-throat in 5 (55.6%), chest tightness in 1 (11.1%), headache and muscle aches in 1 (11.1%), and diarrhea in 1 (11.1%). The median clinical resolution was 3 days (range 1–14). The lung CT scans after infection were normal for all the nine donors. Twenty donors had not yet been infected COVID-19 until completion of apheresis. The baseline and transplant characteristics were similar among there three groups (Table 1).

### Transplant grafts

All the apheresis were successful with a median CD34^+^ cells of 2.33 (2.09–3.01) × 10^6^/kg in CPD, 2.76 (2.02–3.76) × 10^6^/kg in CED, and 2.64 (2.12–3.33) × 10^6^/kg in CND (*p* > 0.05). The median mononuclear cell (MNC) count was 215.64 × 10^9^/L in CPD, 205.62 × 10^9^/L in CED, and 243.42 × 10^9^/L in CND (*p* > 0.05). The median number of collections per participant was 1 (range 1–3). Fifty participants (67.6%) achieved the targeted collection volume with just one collection, 22 participants (29.7%) required two collections, and two participants (2.7%) required three collections.

**Figure 1:**
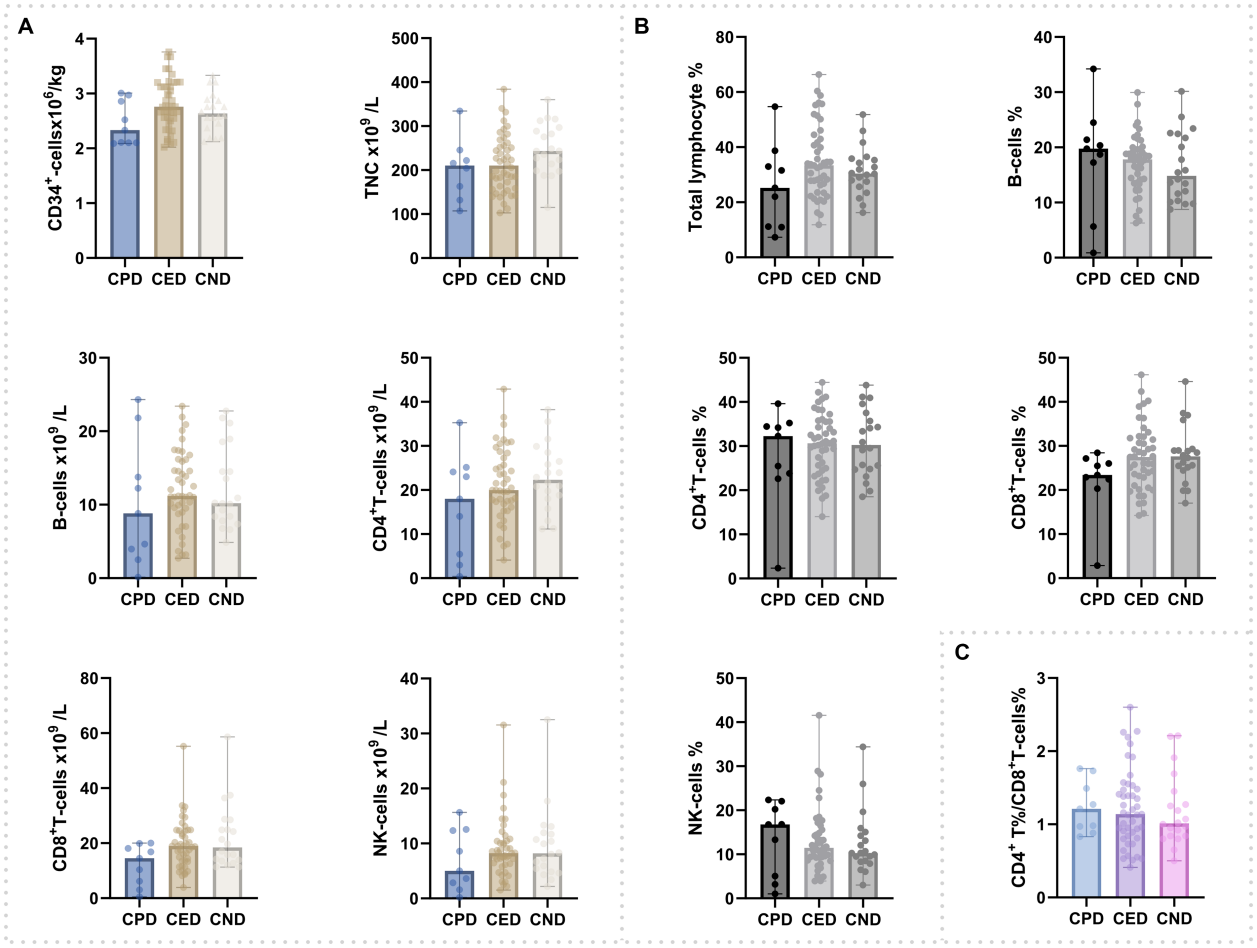
Composition of cells in peripheral blood grafts. **(A)** Counts of CD34^+^cells, total nucleated cells, and lymphocyte subsets. **(B)** Percentage of total lymphocyte and lymphocyte subsets; **(C)** CD4^+^-T%/CD8^+^-T%.

There was no significant difference among three groups regarding lymphocytes [T cells, B cells, and natural killer (NK) cells] (Figure 1; Supplementary Table 1). All the grafts of CPD were tested SARS-CoV-2 negative by RT-PCR.

### Engraftment

All the participants achieved engraftment within 30 days post-transplantation. The median time to neutrophil reconstitution was 12 days (range 9–17), 11 days (range 9–22), and 11 days (range 9–19) in CPD, CED, and CND groups, respectively (*p* > 0.05). The median platelet reconstitution time was 14 days (range 12–29), 12 days (range 7–38), and 12 days (range 8–16) in CPD, CED, and CND groups, respectively (*p* > 0.05). All the participants achieved complete donor chimerism by day30 post-HSCT.

### Graft-versus-host disease

The incidence of II-IV aGVHD within 100 days post-transplant were 55.6% (95% CI, 22.66–84.66%), 20% (95% CI, 10.09–35.05%), and 10% (95% CI, 1.75–33.13%) in the CPD, CED, and CND groups, respectively (*p* = 0.024). The incidence of grade II-IV aGVHD was higher in CPD compared to CND (*p* = 0.016), but similar to CED. In the CPD group, two participants experienced grade II aGVHD and three participants experienced grade III aGVHD. In the CED group, there were four participants with grade II aGVHD, four with grade III aGVHD, and one with grade IV aGVHD (Table 2). Conversely, in the CND group, only two participants developed grade II aGVHD. Univariate analysis indicated that donor being COVID-19-positive at the time of apheresis was a potential risk factor for II-IV aGVHD (*p* = 0.015; OR = 6.136 with a 95% CI of 1.417–26.581). Increased NK cells count (*p* = 0.040; OR = 0.849 with a 95% CI 0.726–0.993) and higher proportion of NK cells in the lymphocyte population (*p* = 0.033; OR = 0.876 with a 95% CI of 0.776–0.990) in the graft were protective factors against II-IV aGVHD in univariate analysis (Table 3). In the multivariable analysis, donor being COVID-19-positive at the time of apheresis appeared to be associated with a heightened risk of II-IV aGVHD (*p* = 0.020; OR = 12.159 with a 95% CI of 1.783–135.760) (Table 3). For participants surviving over 100 days after transplantation, the incidence of cGVHD within 180 days post-transplant was 22.2% (95% CI, 3.95–59.81%), 9.1% (95% CI 2.95–22.58%), and 31.6% (95% CI 13.56–56.50%) in CPD, CED, and CND, respectively (*p* > 0.05). No severe cGVHD was observed (Table 2).

**Table 2:** Comparison of II-IV aGVHD and cGVHD among three groups.

	CPD group (*n* = 9)	CED group (*n* = 45)	CND group (*n* = 20)	*p* value
**II-IV aGVHD (%)**	5 (55.6%)	9 (20.0%)	2 (10.0%)	**0.024**
Grade II	2 (22.2%)	4 (8.9%)	2 (10.0%)	-
Grade III	3 (33.3%)	4 (8.9%)	0	-
Grade IV	0	1 (2.2%)	0	-
**cGVHD (%)**	2 (22.2%)	4 (9.1%)	6 (31.6%)	0.073
Mild	1 (11.1%)	2 (4.5%)	2 (10.5%)	-
Moderate	1 (11.1%)	2 (4.5%)	4 (21.1%)	-

CPD, COVID-19-positive donor; CED, COVID-19-experienced donor; CND, COVID-19-naive donor; and GVHD, Graft-versus-host disease. *p* < 0.05 values are indicated in bold.

**Table 3:** Risk features of II-IV aGVHD post-HSCT by univariate and multivariate logistic regression analysis.

Risk factors	Univariate analysis	*p* value	Multivariate analysis	*p* value
	OR (95%CI)		OR (95%CI)	
**Infection status of donors**				
COVID-19 positive vs. Non-COVID-19 positive	6.136 (1.417–26.581)	**0.015**	12.159 (1.783–135.760)	**0.020**
**Age of recipients**	1.016 (0.970–1.065)	0.499	-	-
**Age of donors**	1.013 (0.973–1.055)	0.531	-	-
**Sex of recipient**				
Male vs. Female	0.871 (0.288–2.636)	0.807	-	-
**Sex of donor**				
Male vs. Female	0.933 (0.308–2.824)	0.903	-	-
**HLA match (%)**				
10 of 10 HLA match vs. 1 or more HLA mismatch(es)	0.913 (0.292–2.857)	0.876	0.629 (0.140–2.507)	0.522
**Conditioning regimen**				
BU-based vs. TBI-based	1.512 (0.378–6.046)	0.559	-	-
**GVHD prophylaxis**				
ATG vs. non-ATG	0.838 (0.251–2.793)	0.774	0.649 (0.138–3.181)	0.581
**CD34^**+**^ × 10^**6**^/kg**	0.682 (0.184–2.530)	0.567	-	-
**MNC × 10^**9**^/L**	1.000 (0.991–1.009)	0.989	-	-
**Total lymphocyte %**	0.974 (0.929–1.021)	0.275	-	-
**B-cells × 10^**9**^/L**	0.985 (0.892–1.087)	0.758	-	-
**CD4^**+**^T-cells × 10^**9**^/L**	0.953 (0.891–1.019)	0.158	-	-
**CD8^**+**^T-cells × 10^**9**^/L**	1.002 (0.948–1.060)	0.930	-	-
**NK-cells × 10^**9**^/L**	0.849 (0.726–0.993)	**0.040**	1.044 (0.807–1.349)	0.740
**B-cells %**	1.007 (0.919–1.104)	0.885	-	-
**CD4^**+**^T-cells %**	0.958 (0.893–1.029)	0.238	-	-
**CD8^**+**^T-cells %**	1.011 (0.937–1.090)	0.784	-	-
**NK-cells %**	0.876 (0.776–0.990)	**0.033**	0.819 (0.626–1.002)	0.097

HLA, Human leukocyte antigen; BU, Busulfan; TBI, Total body irradiation; ATG, Antithymocyte globulin; MNC, Mononuclear cell; NK-cells, Natural Killer cells; B-cells, B lymphocytes; OR, Odds ratio; and CI, Confidence interval. *p* < 0.05 values are indicated in bold.

### Viral infections and other transplant outcomes

One participant in the CPD group suffered COVID-19 at day 25 after transplant. Twelve participants suffered COVID-19 in the CND group with a median of 59.5 days (range 13–160) post-transplant, while eight had COVID-19 in the CED group with 39.5 days (range 25–251).

The cumulative incidence of EBV viremia within 180 days post-transplant was 11.1% (95% CI 0.58–49.33%), 20.0% (95% CI 10.09–35.05%), and 30.0% (95% CI 12.84–54.33%) in CPD, CND, and CED groups, respectively (*p* > 0.05). The corresponding median time to onset of EBV viremia was 25 days, 44 days (range 23–91 days), and 42.5 days (range 25–58 days) post-transplantation. The cumulative incidence of CMV viremia was 33.3% (95% CI 9.04–69.08%), 31.1% (95% CI 18.62–46.80%), and 35.0% (95% CI 16.31–59.05%) in in CPD, CND, and CED groups, respectively (*p* > 0.05). The corresponding median time to onset of CMV viremia was 29 days (range 29–44 days), 34 days (range 23–102 days), and 32 days (range 20–88 days) post-transplantation. For recipients infected with COVID-19 after transplantation, only one participant in the CND group suffered EBV viremia within 2 weeks after COVID-19 infection. None of these participants developed CMV disease or EBV-associated lymphoproliferative disorder.

Sinusoidal Obstruction Syndrome (SOS) was observed in two of the 45 CED-participants (4.4%) and in one of the 20 CND-participants (5.0%). Three participants developed mild to moderate hemorrhagic cystitis.

### Survival and relapse

**Figure 2:**
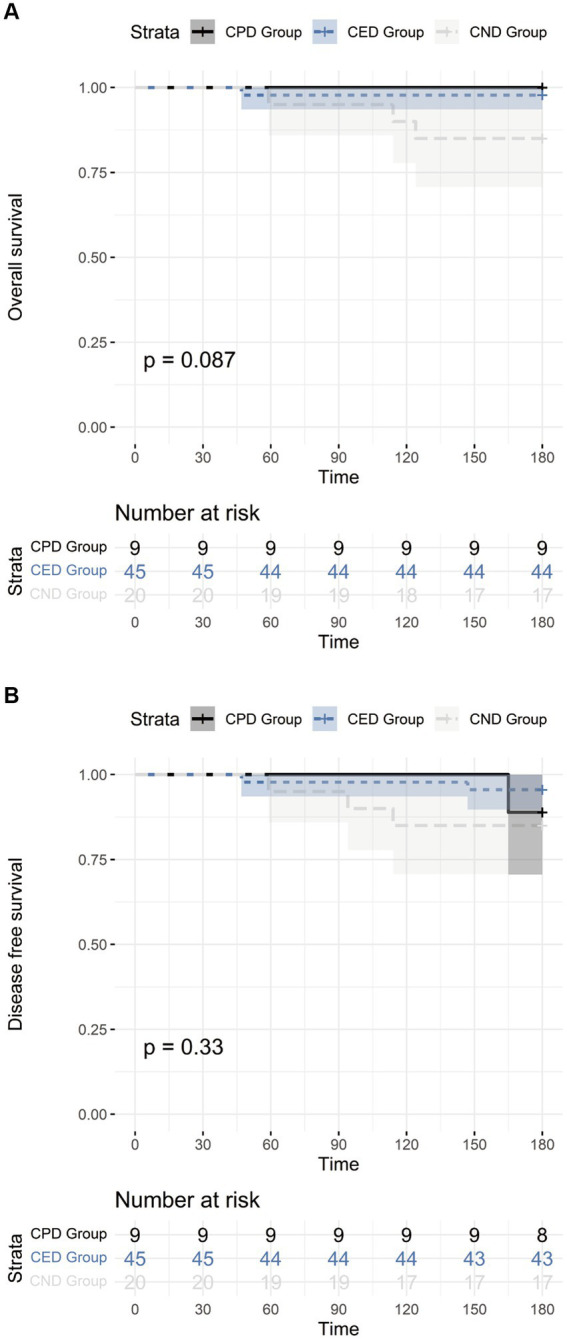
**(A)** The overall survival of CPD, CED, and CND groups; **(B)** The disease-free survival of CPD, CED, and CND groups.

The median follow-up was 255 days (IQR 226–319 days). Four participants died within the follow-up period including one in CED group and three in CND group. The causes of death included severe pneumonia (*n* = 2), aGVHD (*n* = 1), and leukemia relapse (*n* = 1). The OS at 6 months post-transplant was 100.0% (95% CI 62.88–100.00%), 97.8% (95% CI 86.77–99.88%), and 85.0% (95% CI, 61.14–96.04%) in CPD, CED, and CND groups, respectively (*p* > 0.05) (Figure 2A). Primary disease relapse occurred in two participants (one in CPD and one in CEP group) with DFS at 6 months of 88.9% (95% CI 50.67–99.42%), 95.6% (95% CI 83.64–99.23%), and 85.0% (95% CI 61.14–96.04%) in CPD, CED, and CND groups, respectively (*p* > 0.05) (Figure 2B).

## Discussion

SARS-CoV-2 is transmissible and often leads to varying degrees of alterations in peripheral blood lymphocyte counts and composition (Klein et al., 2023; Qin et al., 2023). Our multicenter retrospective study evaluated the safety and efficacy of apheresis in COVID-19 positive donors. Moreover, we explored the disparities in graft characteristics and outcomes of allo-HSCT recipients receiving transplantation with COVID-19 current-positive, previously positive, and negative donors during COVID-19 pandemic.

The safety of COVID-19 positive donor is main concern during mobilization and apheresis. Several previous studies have described clinical cases about HSCT donor with COVID-19 at the time of collection, and none of these donors were reported to have developed severe infection (Blandin et al., 2022; Lin et al., 2023). In the current study, none of the 9 COVID-19 positive donors deteriorated into moderate or severe infection, which indicates that mobilization and apheresis are safe in COVID-19 positive donors.

It remains undetermined whether SARS-CoV-2 can be transmitted through blood products. The theoretical risk of SARS-CoV-2 transmission through blood products comes from the observation of SARS-CoV-2 RNAemia in patients with COVID-19 (Huang et al., 2020; Li et al., 2021). Several studies reported the detection of SARS-CoV-2 RNA by RT-PCR in blood products, including plasma and lymphocytes, from a very small number of positive blood donors (Wang et al., 2004; Cappy et al., 2020; Huang et al., 2020). However, no positive SARS-COV-2 detection in the CPD grafts in our study. Additionally, none of the nine recipients in the CPD group exhibited symptoms of COVID-19 except 1 suffered COVID-19 at +25 days post-transplant. Consistent with our findings, Anurathapan et al. (2020) and Blandin et al. (2022) reported cases of individuals receiving allo-HSCT from COVID-19 positive donors, with grafts free of SARS-CoV-2, and no COVID-19 symptoms observed in recipients after transplantation. Additionally, several previous studies demonstrated that allo-HSCT recipients showed no COVID-19 symptoms after receiving platelets and mobilized peripheral blood stem cells from COVID-19 positive donors (Essa et al., 2020; Mawalla et al., 2021). Therefore, the risk of SARS-CoV-2 transmission from graft should be further studies.

Considering the potential effect of COVID-19 on grafts (Huang et al., 2020), we compared CD34^+^ cells and MNC of grafts derived from COVID-19 positive, experienced and negative donors. No differences were observed among the three groups. Consistent with our results, a study with 16 COVID-19 positive donor and 55 COVID-19 negative donor (Lin et al., 2023) reports that CD34^+^ and MNC dose were comparable among these participants. Either this study or ours did not observe the effect of graft components on engraftment. Successful engraftment is also documented in previous reports involving recipients who received grafts from COVID-19 positive donors (Anurathapan et al., 2020; Cheng et al., 2023; Lin et al., 2023).

Lymphopenia has been reported to occur in up to 80–90% of the participants with COVID-19 (Huang et al., 2020; Yang et al., 2020). In the current study, no significant differences were found either in donors’ lymphocytes at the day of apheresis or lymphocytes in grafts among the three donor groups. Unexpectedly, our results showed that the incidence of grade II–IV aGVHD was higher in the recipients receiving HSCT from COVID-19 positive donors than those receiving HSCT from non-COVID-19 positive donors. There are limited published studies that focused specifically on this issue (Cheng et al., 2023). Consistent with our results, Li et al. also found that the incidence of grade II-IV aGVHD and III-IV aGVHD among donor-COVID-positive recipients were higher, compared to donor-COVID-negative recipients, although the difference was not statistically significant (Lin et al., 2023). Activated T cells, including the CD4^+^ T, CD8^+^ T, and Th cells, were found enriched in COVID-19 participants (Rupp et al., 2021; Qin et al., 2023). Moreover, cytokine levels were shown to be higher in COVID-19 participants (Hasanvand, 2022; Queiroz et al., 2022; Tiwari et al., 2023). As aGVHD is mediated by the activation of alloreactive T cells with high level of cytokine and chemokine (Lange et al., 1995; Zeiser and Blazar, 2017; Khandelwal et al., 2020), we presume that increased aGVHD in the CPD group might be attributed to the active T cells and cytokines in graft derived from COVID-19-positive donors.

For other complications of transplant, we observed no difference among the three groups. In addition, donors’ COVID-19 status at transplant did not affect OS or DFS within +6 months post-transplant. Consistent with our findings, Li et al. found no significant difference in viral infection or survival between COVID+ donor and COVID-donor recipients (Lin et al., 2023).

This study has several limitations. The sample size is relatively small and the follow-up period is relatively short. The functional subgroup of lymphocyte in grafts is not investigated for a better understanding of increased aGVHD in CPD.

In conclusion, our study indicates that the COVID-19-positive status of donors does not impact graft collection, engraftment, or short-term survival of participants. However, it may pose a potential risk factor for the incidence of grades II-IV aGVHD. Further follow-up is required to determine the long-term effects of donor’s COVID-19 status on transplant outcomes.

## Data availability statement

The original contributions presented in the study are included in the article/Supplementary material; further inquiries can be directed to the corresponding authors.

## Ethics statement

The studies involving humans were approved by Medical Ethics Committee of Nanfang Hospital of Southern Medical University. The studies were conducted in accordance with the local legislation and institutional requirements. The participants provided their written informed consent to participate in this study.

## Author contributions

YifH: Data curation, Methodology, Visualization, Writing – original draft. ZF: Writing – original draft. YinH: Writing – original draft. SF: Writing – original draft. SW: Writing – original draft. SZ: Writing – original draft. FH: Writing – original draft. LX: Writing – original draft. NX: Writing – original draft. HL: Writing – original draft. ZW: Writing – original draft. JS: Writing – original draft. QL: Supervision, Writing – review & editing. RL: Supervision, Writing – review & editing, Funding acquisition.

## Glossary

CPD: COVID-19-positive donor
CED: COVID-19-experienced donor
CND: COVID-19-naive donor
AML: Acute myeloid leukemia
ALL: Acute lymphoblastic leukemia
AA: Aplastic anemia
MDS: Myelodysplastic syndrome
MPAL: Mixed-phenotype acute leukemia
PB: Peripheral blood
UCB: Umbilical cord blood
HLA: Human leukocyte antigen
BU: Busulfan
TBI: Total body irradiation
ATG: Antithymocyte globulin
allo-HSCT: Allogeneic hematopoietic stem cell transplantation
GVHD: Graft-versus-host disease
aGVHD: Acute GVHD
cGVHD: Chronic GVHD
EBV: Epstein–Barr virus
CMV: Cytomegalovirus
MNC: Mononuclear cell
NK-cells: Natural Killer cells
B-cells: B lymphocytes
SOS: Sinusoidal obstruction syndrome
OS: Overall survival
DFS: Disease-free survival
NAT: Nucleic acid amplification test
RT-PCR: Reverse transcription PCR
