# Supplementary material for: The impact of the donors’ COVID-19 status on the outcomes of allogeneic hematopoietic stem cell transplantation: a multi-center retrospective study
Source: Front Microbiol. 2024 Jul 15;15:1415289. doi: 10.3389/fmicb.2024.1415289 (PMC11284148; doi:10.3389/fmicb.2024.1415289)
Supplement: Supplementary file 1 [file Table_1.DOCX]

**Supplementary Table 1. Composition of immune cells in peripheral blood grafts**

| **Parameters**  **(median)** | **CPD- group**  **（n=9）** | **CED- group**  **（n=45）** | **CND- group**  **（n=20）** | **p-value** |
| --- | --- | --- | --- | --- |
| **Median absolute value (10^9^/L, range)** |  |  |  |  |
| TNC | 215.64 (107.39-334.40) | 205.62 (102.54-383.86) | 243.42 (115.40-360.29) | 0.081 |
| B-cells | 8.81 (0.15-24.31) | 11.19 (2.17-23.42) | 10.21 (4.86-22.77) | 0.566 |
| CD4^+^T-cells | 17.97 (0.40-35.29) | 19.99 (4.09-42.90) | 22.32 (11.13-38.23) | 0.353 |
| CD8^+^T-cells | 14.49 (0.48-20.00) | 18.96 (3.92 -55.22) | 18.41(11.29-58.63) | 0.081 |
| NK-cells | 5.03 (0.17- 15.66) | 8.23 (1.51-31.56) | 8.18 (2.18-32.53) | 0.582 |
| **Median percentage (%, range)** |  |  |  |  |
| Total lymphocyte | 25.25 (7.30-54.69) | 33.39 (11.18-66.38) | 30.45 (16.23-51.85) | 0.207 |
| B-cells | 19.74 (0.89-34.21) | 17.81 (6.27-29.96) | 14.83 (8.75-30.15) | 0.579 |
| CD4^+^T-cells | 32.29 (2.36-39.59) | 30.64 (14.00-44.43) | 30.21 (18.53%-43.81) | 0.950 |
| CD8^+^T-cells | 23.38 (2.84-28.45) | 27.49 (14.22-46.16) | 27.59 (17.02-44.61) | 0.176 |
| NK-cells | 16.78 (1.03-22.36) | 11.49 (3.95-41.58) | 10.29 (3.03-34.38) | 0.739 |
| **CD4^+^T-cells% / CD8+T-cells% range** | 1.21 (0.83-1.76) | 1.14 (0.41-2.60) | 1.01(0.50-2.21) | 0.797 |

Abbreviations: CPD, COVID-19-positive donor; CED, COVID-19-experienced donor; CND, COVID-19-naive donor; TNC, Total Neutrophil Count; NK-cells, natural Killer cells; B-cells, B lymphocytes;
